# The Emperor Has No Symptoms: The Risks of a Blanket Approach to Using Epinephrine Autoinjectors for All Allergic Reactions

**DOI:** 10.1016/j.jaip.2016.05.005

**Published:** 2016

**Authors:** Paul J. Turner, Audrey DunnGalvin, Jonathan O'B. Hourihane

**Affiliations:** a Section of Paediatrics (Allergy and Infectious Diseases) and Medical Research Council and Asthma UK Centre in Allergic Mechanisms of Asthma, Imperial College London, London, United Kingdom; b Division of Paediatrics & Child Health, University of Sydney, New South Wales, Australia; c School of Applied Psychology, University College Cork, Cork, Ireland; d Paediatrics and Child Health, University College Cork, Cork, Ireland

**Keywords:** Food allergy, Anaphylaxis, Epinephrine, Autoinjector, Emergency management, ED, Emergency departments, EMS, Emergency medical services

## Abstract

Fatal anaphylaxis in humans is rare and unpredictable. We note a trend to provide allergic individuals with care plans that recommend immediate use of epinephrine autoinjectors if allergen ingestion is suspected, even in the absence of any allergic symptoms, without any supporting evidence base. Instructions to use an autoinjector device, irrespective of reaction severity and especially when symptoms are *actually* absent, are likely to add to parental and patient anxiety. Of greater concern is the possibility of epinephrine being administered “too early” to treat initial, mild symptoms that then progress to severe anaphylaxis. It is not hard to visualize a scenario where one or both epinephrine autoinjectors have been deployed for mild symptoms, yet the reaction progresses to a severe reaction and no further epinephrine is available for administration. Epinephrine needs to be available as a rescue treatment for anaphylaxis, potentially buying valuable minutes while emergency medical services are activated to attend. Food-allergic individuals and their carers need to be provided with more constructive strategies and support than merely being told to “use your pen.”

A new emergency care plan for food-allergic individuals was published by the Food Allergy Research & Education (FARE) earlier this year. We find some elements in the plan^1^ controversial, and are unaware of the evidence base on which some of the directives (Figure 1) have been promulgated, particularly the recommendation to administer epinephrine even in the absence of symptoms.

It is very difficult to determine whether an allergic individual is “extremely reactive” or not. The term “reactivity” causes confusion: does it refer to dose sensitivity (or threshold) to allergen, or severity of the elicited reaction? Most individuals who react to food allergens at the bottom of the dose response curve (derived from food challenge data) experience only mild reactions.^2^, ^3^ Furthermore, data suggest that individuals with a history of anaphylaxis are not more dose sensitive than those without a history of anaphylaxis.^4^, ^5^, ^6^, ^7^ The vast majority of fatal reactions are due to substantial levels of exposure accidentally ingested, often with other significant cofactors present contributing to the severity of the reaction.^8^, ^9^, ^10^

Anaphylaxis to food is not uncommon^11^; however, fatal food-induced anaphylaxis is very rare, with a case fatality rate at <0.0001%.^12^, ^13^ Data suggest that, in many cases, anaphylaxis (admittedly at the less severe end of the anaphylaxis spectrum) resolves without any treatment^14^—a daily event in many emergency departments (ED), where patients present having experienced symptoms of anaphylaxis but failed to use their autoinjector or have not had their anaphylaxis correctly diagnosed in the ED. Arguably, our biggest challenge in the management of food-allergic individuals is in identifying those who are most at risk of severe, life-threatening reactions; although fatal anaphylaxis is rare, it is also very unpredictable.^11^ As a result, we have to apply the same management strategies to all food-allergic individuals, resulting in the widespread provision of rescue medication including epinephrine autoinjectors to anyone considered to be at risk of anaphylaxis. That in itself is a further challenge; the limited published data imply that up to 3 quarters of peanut-allergic children will have anaphylaxis if exposed to *sufficient* allergen.^15^

## Can Using Epinephrine Autoinjectors for Mild Allergic Symptoms Cause Harm?

We believe that the use of epinephrine for *any* exposure to an allergen is overtreatment, and may, paradoxically, place some individuals at greater risk of severe outcomes. The concept of “very early” epinephrine, given immediately at the onset of any symptom seems to be overinvasive medical advice, which has implications for negative patient perception of the burden in managing daily life with this condition. One can argue that given the difficulty for patients in identifying their own symptoms of anaphylaxis,^16^, ^17^ it may simplify management by advising epinephrine to be administered for *all* reactions. However, given the tendency of patients, and their parents, *not* to use epinephrine autoinjectors even when appropriate to do so^14^—often out of anxiety for the consequences of an injection and/or underlying needle phobia—we would be concerned that a lowering of the threshold to give epinephrine would only increase the reluctance of individuals to administer an injection, even when it is warranted. Similar to the widespread use of precautionary allergen labelling on food packaging, blanket strategies often give rise to greater, rather than less risky behaviors.

We are concerned that a recommendation to use epinephrine autoinjectors for *any* symptoms of an allergic reaction might be driven, in part, by commercial interests. The patient literature provided by at least one manufacturer of epinephrine autoinjectors appears to imply that the device should be used for “critical symptoms,” which according to the instructions include mouth swelling and skin symptoms.^18^ This trend or “slippage”—where nonsevere symptoms are labeled “critical”—is concerning; guidelines and patient literature should be written by nonconflicted individuals with appropriate expertise, and not driven by a marketing agenda. From a patient perspective, this may further compound confusion over what constitutes “anaphylaxis,” giving rise to stress and anxiety, particularly for parents of young children.^19^, ^20^

Delayed use of epinephrine in evolving anaphylaxis has been associated with adverse outcomes including death.^8^, ^9^ Epinephrine is generally well tolerated by most individuals, even children, who receive it intramuscularly,^21^, ^22^ and, of course, we do encourage our patients to use their epinephrine autoinjector if they have signs of airway/respiratory or cardiovascular involvement, or if they are uncertain what to do, because of difficulty in recognizing or identifying symptoms in themselves or in their child. We also advocate epinephrine use in the context of a rapidly progressing reaction. However, we do *not* advise patients to inject epinephrine for urticaria or angioedema, because, if they occur in isolation or rather in the absence of other symptoms or signs, they are nearly always minor self-limiting symptoms that, in our view, do not justify epinephrine injection. One also has to consider that tremor and/or shaking is not uncommon after epinephrine autoinjector use^22^; this can be justified in the context of treating anaphylaxis, but if used for mild reactions, the allergic individual may develop a reluctance to use epinephrine that impacts on the decision to treat subsequent reactions—including anaphylaxis—with appropriate rescue medication.

Food-allergic individuals often receive differing advice on their level of reactivity from clinicians, who themselves have differing levels of expertise and experience of food allergy and anaphylaxis, which, incidentally, are not the same thing.^23^ A food-allergic individual's perception of his and/or her own risk—and likelihood of reaction—may differ from that of an experienced clinician. Parents are understandably motivated to protect their own child to the greatest possible extent in their role as parents, but the allergy literature is now full of reports of disproportionate parental anxiety impacting adversely on family life, interpersonal relationships, and general social functioning.^19^, ^20^, ^24^ Instructions to use an autoinjector device, irrespective of severity and especially when symptoms are *actually* absent, are likely to add to parental and patient anxiety; this is maladaptive and counterproductive, in that it results in lower self-efficacy and competence in emergency care,^25^, ^26^ in turn leading to a greater adverse impact on food allergy-related quality of life.

The idea that consumption of any amount of allergen (eg, peanut) should motivate immediate administration of epinephrine, even when no symptoms have been elicited, is very controversial. Up to 50% of challenge-proven peanut-allergic individuals do not react to doses up to 100 mg of peanut protein (about half a peanut),^3^, ^4^, ^5^ although we acknowledge that the formal challenge scenario is an artificial setting and that community reactions may involve other covariables. In the panic caused by possible exposure, it can be difficult to discern whether true exposure has occurred. If a peanut-allergic individual has inadvertently eaten a candy bar and only then notices a “may contain” warning, should that individual be instructed to self-administer epinephrine? Almost two thirds of prepacked confectionery are labeled with some form of precautionary allergen label.^27^ The majority of nut-allergic individuals will eat some of these products.^28^, ^29^ A blanket policy of epinephrine administration in the absence of any symptoms is a step too far.

Our biggest concern is that the data from case series of fatal food-induced anaphylaxis have reported a median time to cardiorespiratory arrest (from consumption of the allergen) of around 30 minutes.^30^ The plasma half-life of epinephrine is 2-3 minutes, although intramuscular injection prolongs this. It is therefore conceivable that epinephrine administered “too early” may limit management options if used to treat initial, mild symptoms that then progress (with gastrointestinal absorption) to severe anaphylaxis. Epinephrine autoinjectors are frequently used incorrectly^31^, ^32^ or have failed for technical reasons, resulting in epinephrine not being administered; this is likely to have contributed to fatal outcomes in some cases.^9^ It is not hard to visualize a scenario where one or both epinephrine autoinjectors have been deployed for mild symptoms, yet the reaction progresses to a severe reaction and no further epinephrine is available for administration. This scenario may be even more likely to occur in the context of an individual administering the autoinjector to a child. One might argue that because users are advised to contact emergency medical services (EMS) after the use of an autoinjector, a lack of further epinephrine autoinjectors is less of an issue. However, in practice, allergic individuals do not always contact EMS in a timely manner, if at all^33^; where the health system levies a charge for EMS, this may not always be covered by health insurance and may thus provide a further disincentive to activate EMS.^34^ Furthermore, EMS response times vary, with *average* times from initial contact to arrival on scene of around 9 minutes in the USA^35^ and Europe.^36^ Thus, 50% of response times are longer than this, and these times do not include time to summon EMS nor the time taken to locate the patient after arrival at the address provided to EMS. It is therefore entirely feasible that there will be occasions when EMS support is delayed, and thus further epinephrine is not available in the context of an ongoing anaphylactic reaction.

There are no human reports or studies using animal models that show that “early” or “hyper-soon” epinephrine aborts what could—at least in animal studies—be considered inevitable or certain anaphylaxis. Indeed, there is at least one case report of a patient who was repeatedly administered epinephrine during an initially mild allergic reaction to brazil nut, which did not prevent progression to fatal respiratory arrest.^30^ Furthermore, up to one third of fatalities due to food-induced anaphylaxis are administered at least one dose of epinephrine in a timely manner, and yet the outcome is still death.^8^, ^9^ We note that the fatality rate for cases of food-induced anaphylaxis admitted to intensive care units for cardiorespiratory intervention (ie, artificial ventilation and inotropic support) is very low.^37^ Severe anaphylaxis will respond to intensive management, and self-administered epinephrine is a critical intervention to prolong the window of opportunity for this to occur. Premature administration of epinephrine may be counterproductive, reducing the time available for medical intervention.

Early use of epinephrine autoinjectors may increase the rates of adverse outcomes (by increasing the numbers of autoinjector devices actually deployed); this could impair public and medical confidence in evidence-based self-care plans, and even in the advice and advocacy provided by health care professionals. Presumably, the advice we are contesting here is a well-meant blanket policy, to protect those who may not receive sufficient and appropriate education in understanding and implementing expert-developed, evidence-based self-care plans. Such patients, and those caring for them, are likely to be “deficient” in self-efficacy—less able to manage an allergic reaction appropriately, and advised to overtreat themselves with a medical device they may have been inexpertly coached to use properly—all of which makes misuse more likely. “Crying wolf” may become an issue; “cases” that are treated—but were never “cases” at all—may diminish first-responder reactions to “cases” that are “real.” This is not just hypothetical; in the UK, there are ongoing concerns as to whether the EMS response to anaphylaxis will be downgraded in priority,^38^ a situation likely to be exacerbated with “hyper-soon” epinephrine use.

## Conclusions

In humans, where fatal anaphylaxis is rare and unpredictable, we contend that a care plan that recommends the use of epinephrine autoinjectors, in the absence of *any* symptoms of an allergic reaction, is ill-advised and not based on any supporting evidence. Epinephrine needs to be available as a rescue treatment for anaphylaxis, potentially buying valuable minutes while EMS are activated to attend. Food-allergic individuals and their carers need to be provided with more constructive strategies and support than merely being told to “use your pen.”

## Figures and Tables

**Figure 1:**
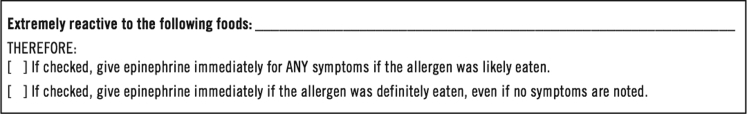
Extract from the Food Allergy Research & Education (FARE) emergency self-care plan.^1^
